# Prospective study of weight loss and all-cause-, cardiovascular-, and cancer mortality

**DOI:** 10.1038/s41598-023-32977-8

**Published:** 2023-04-06

**Authors:** Liisa Tolvanen, Francesca Ghilotti, Hans-Olov Adami, Weimin Ye, Stephanie E. Bonn, Rino Bellocco, Ylva Trolle Lagerros

**Affiliations:** 1 Division of Clinical Epidemiology, Department of Medicine Solna, Karolinska Institutet, Stockholm, Sweden; 2 Center for Obesity, Academic Specialist Center, Stockholm Health Care Services, Stockholm, Sweden; 3 Department of Medical Epidemiology and Biostatistics, Karolinska Institutet, Stockholm, Sweden; 4 Clinical Effectiveness Group, Institute of Health and Society, University of Oslo, Oslo, Norway; 5 Department of Statistics and Quantitative Methods, University of Milano-Bicocca, Milan, Italy

**Keywords:** Cardiology, Risk factors

## Abstract

Effects of repeated weight changes on mortality are not well established. In this prospective cohort study, we followed 34,346 individuals from 1997 to 2018 for all-cause mortality, and 2016 for cause-specific mortality. At baseline, participants self-reported amount and frequency of prior weight loss. During 20.6 (median) years of follow-up, we identified 5627 deaths; 1783 due to cancer and 1596 due to cardiovascular disease (CVD). We used Cox Proportional Hazards models to estimate multivariable‐adjusted Hazard Ratios (HRs) and 95% confidence intervals (CI). Participants with a weight loss > 10 kg had higher rates of all-cause (HR 1.22; 95%CI 1.09–1.36) and CVD mortality (HR 1.27; 95%CI 1.01–1.59) compared to individuals with no weight loss. Men who had lost > 10 kg had higher all-cause (HR 1.55; 95%CI 1.31–1.84) and CVD mortality (HR 1.55; 95%CI 1.11–2.15) compared to men with no weight loss. Participants who had lost ≥ 5 kg three times or more prior to baseline had increased rates of all-cause (HR 1.16; 95%CI 1.03–1.30) and CVD mortality (HR 1.49; 95%CI 1.20–1.85) compared to participants with no weight loss. We found no association between weight loss and cancer mortality. We conclude that previous and repeated weight loss may increase all-cause and CVD mortality, especially in men.

## Introduction

The global epidemic of overweight and obesity is of growing concern for the general population, public health, and healthcare professionals due to its profound health consequences. According to the World Health Organization^1^, the global prevalence of obesity has nearly tripled since the mid-1970s. Intentional weight loss in people with overweight and obesity may, however, improve health and reduce the risk of co-morbidities and mortality^2^. A weight loss of 5 to 10% is considered clinically significant and can result in improved metabolic parameters such as blood glucose, blood pressure, and lipid profiles^3^.

Behavioral modification combined with dietary- and physical activity interventions, as investigated in the Look Ahead (Action for Health in Diabetes) study, led to a weight loss of almost 5% at the eight years of follow-up among persons with overweight or obesity and type 2 diabetes^4^. Anti-obesity medication may additionally increase weight loss^5^, while bariatric surgery leads to the most sustainable weight loss over time in patients with severe obesity^6^. Nevertheless, achieving sustainable weight loss is demanding, and weight regain often occurs regardless of treatment method, also after anti-obesity medication^7^ and bariatric surgery^8^.

Weight changes (gain, loss, and regain) are affected by complex interacting genetic, environmental, behavioral, socioeconomic, psychosocial, and medical factors^9^. Although repeated weight change (weight cycling) has been associated with increased mortality^10–12^, available evidence is inconclusive because some studies do not support an association between repeated weight changes and increased mortality^13,14^.Women are engaged in weight loss attempts more often than men^15^. Therefore, an improved understanding of the effects of weight loss and repeated weight loss among men and women on all-cause mortality, cardiovascular (CVD), and cancer mortality is essential for developing evidence-based clinical recommendations. To this end, we undertook a large prospective study with more than 20 years of a virtually complete follow-up in national registries which offered a possibility to quantify the association between previous weight loss and repeated weight loss with all-cause, CVD, and cancer mortality in women and men.

## Results

### Baseline characteristics

Baseline characteristics by categories of weight loss are presented in Table 1. Nearly half of the participants (46.5%) reported a weight loss of 5 kg or more (51.7% of women and 36.7% of men). Of those, 51.2% reported having lost weight once, 23.8% twice, and 25.0% three times or more. Participants who had lost 5 to 10 kg at least once had a mean body mass index (BMI) at baseline of 25.0 kg/m^2^, and those who had lost > 10 kg at least once, had a mean BMI of 27.3 kg/m^2^. Participants who did not report any weight loss were on average 2 years older than those who had lost weight. They were also more likely to have longer sleep duration, less likely to smoke and they rated their health better than those who had lost weight. Further, participants in the highest weight loss category (> 10 kg) were more likely to have a low income and be less physically active than those who reported no weight loss or a weight loss of 5 to 10 kg. The prevalence of central obesity—defined by a waist circumference of more than 88 cm among women, and 102 cm among men—was 12.6% among participants with no weight loss, 23,5% among those who reported a weight loss of 5 to 10 kg and 42.9% among those reporting a weight loss > 10 kg. Up to April 2018, 5627 deaths occurred (4844 up to December 2016). The main causes of death were cancer (n = 1783) and CVD (n = 1596).

**Table 1:** Baseline characteristics of study participants by categories of prior weight loss (n = 34,346).

Characteristics	Weight loss category					
	No weight loss		5–10 kg		> 10 kg	
Number of participants, n (%)	18,370 (53.5)		11,686 (34.0)		4290 (12.5)	
Sex, n (%)						
Female	10,853 (59.1)		8305 (71.1)		3317 (77.3)	
Male	7517 (40.9)		3381 (28.9)		973 (22.7)	
Age (years), mean (SD)	50.4 (16.5)		48.5 (15.2)		48.3 (13.3)	
BMI, body mass index (kg/m^2^), mean (SD)	23.8 (2.8)		25.0 (3.3)		27.3 (4.4)	
BMI, body mass index (kg/m^2^), n (%)						
Normal weight, ≤ 24.9 kg/m^2^	12,618 (71.4)		6133 (54.9)		1366 (33.6)	
Overweight, 25–29.9 kg/m^2^	4607 (26.0)		4207 (37.6)		1729 (42.5)	
Obesity, ≥ 30 kg/m^2^	457 (2.6)		842 (7.5)		974 (23.9)	
Missing (n)	688		504		221	
Waist circumference (cm), n (%)						
< 80 cm female or < 94 cm male	8310 (59.4)		3916 (45.3)		941 (30.5)	
≥ 80–88 cm female or ≥ 94–102 cm male	3907 (28.0)		2696 (31.2)		822 (26.6)	
> 88 cm female or > 102 cm male	1768 (12.6)		2029 (23.5)		1323 (42.9)	
Missing (n)	4385		3045		1204	
Smoking, n (%)						
Never	11,913 (69.8)		6546 (61.2)		2079 (52.9)	
Former	4082 (23.9)		3172 (29.6)		1355 (34.5)	
Current	1073 (6.3)		986 (9.2)		499 (12.7)	
Missing (n)	1302		982		357	
Alcohol consumption, n (%)						
None	2016 (11.0)		1193 (10.3)		516 (12.1)	
Low ≤ 4 times/month	8744 (47.9)		5735 (49.3)		2208 (51.7)	
Medium/high at least once a week	7512 (41.1)		4692 (40.4)		1546 (36.2)	
Missing (n)	98		66		20	
Education, n (%)						
Compulsory school	4163 (22.7)		2355 (20.2)		815 (19.0)	
High school	7880 (43.0)		5161 (44.3)		2036 (47.6)	
University/PhD	6271 (34.3)		4140 (35.5)		1427 (33.4)	
Missing (n)	56		30		12	
Income (annual)^a^, n (%)						
1st tertile ≤ 93,500 SEK	6218 (33.9)		4058 (34.8)		1547 (36.1)	
2nd tertile 93,600–129,000 SEK	6059 (33.0)		3696 (31.6)		1437 (33.5)	
3rd tertile ≥ 129,100 SEK	6081 (33.1)		3928 (33.6)		1302 (30.4)	
Missing (n)	12		4		4	
Physical activity, MET h/day, n (%)						
Low, ≤ 32 female or ≤ 34 male	5834 (31.9)		3650 (31.3)		1399 (32.6)	
Medium, 32 to 38 female or 34 to 46 male	6249 (34.1)		3968 (34.0)		1444 (33.7)	
High, > 38 female or > 46 male	6232 (34.0)		4045 (34.7)		1442 (33.7)	
Missing (n)	55		23		5	
Sleep duration, n (%)						
≤ 6 h	4978 (28.5)		3709 (33.1)		1580 (38.5)	
7 h	7938 (45.4)		4863 (43.3)		1598 (38.9)	
≥ 8 h	4552 (26.1)		2650 (23.6)		928 (22.6)	
Missing (n)	902		464		184	
Subjective health, n (%)						
Good/very good	15,848 (88.1)		9501 (83.0)		3238 (77.4)	
Neither good nor bad	1922 (10.7)		1659 (14.5)		760 (18.2)	
Bad/very bad	212 (1.2)		291 (2.5)		186 (4.4)	
Missing (n)	388		235		106	

*MET* metabolic energy turnover.

^a^Information was retrieved from the Lisa Database in 1997. SEK is the currency code for the Swedish krona.

### Weight loss and all-cause mortality

Mortality rates and HRs for the association between weight loss and all-cause mortality are presented in Table 2. We found a 9% (HR 1.09; 95% CI 1.02–1.17) higher rate of all-cause mortality among those with a reported weight loss of 5 to 10 kg and a 22% (HR 1.22; 95% CI 1.09–1.36) higher rate of all-cause mortality among those with a weight loss > 10 kg compared to participants with no weight loss. The association remained similar when we excluded the first two years of follow-up (Table S1) and fitted models based on multiple imputation (Table S3).

**Table 2:** Mortality rates and hazard ratios for the association between weight loss and overall mortality.

	Weight loss			*P* trend
	No weight loss	5–10 kg	> 10 kg	
All-cause mortality				
Total				
Deaths (n)	3333	1699	595	
Person-years	351,556	226,037	83,578	
Crude mortality rate per 100,000 person-years	948.1	751.6	711.9	
HR (95% CI)^a^	1.00 (reference)	1.13 (1.07–1.20)	1.34 (1.23–1.47)	< 0.001
HR (95% CI)^b^	1.00 (reference)	1.09 (1.02–1.17)	1.22 (1.09–1.36)	< 0.001
Female				
Deaths (n)	1542	1021	374	
Person-years	211,401	162,627	65,419	
Crude mortality rate per 100,000 person-years	729.4	627.8	571.7	
HR (95% CI)^c^	1.00 (reference)	1.11 (1.02–1.20)	1.26 (1.12–1.41)	< 0.001
HR (95% CI)^d^	1.00 (reference)	1.02 (0.93–1.13)	1.05 (0.90–1.21)	0.50
Male				
Deaths (n)	1791	678	221	
Person-years	140,155	63,409	18,158	
Crude mortality rate per 100,000 person-years	1277.9	1069.2	1217.1	
HR (95% CI)^c^	1.00 (reference)	1.16 (1.06–1.26)	1.51 (1.31–1.74)	< 0.001
HR (95% CI)^d^	1.00 (reference)	1.17 (1.05–1.30)	1.55 (1.31–1.84)	< 0.001

*HR* hazard ratio, *CI* confidence interval.

^a^Adjusted for age and sex at enrollment.

^b^Adjusted for age, sex, body mass index, cigarette smoking, alcohol consumption, level of education, income, physical activity, sleep, and subjective health.

^c^Adjusted for age at enrollment.

^d^Adjusted for age, body mass index, cigarette smoking, alcohol consumption, level of education, income, physical activity, sleep, and subjective health.

We found a significant (p-value < 0.01) interaction between sex and weight loss. The association between weight loss and all-cause mortality was apparent in men only. In women, we did neither find a significant association between weight loss of 5 to 10 kg (HR 1.02; 95% CI 0.93–1.13), nor between weight loss of > 10 kg and all-cause mortality (HR 1.05; 95% CI 0.90–1.21) compared to those who reported no weight loss (Table 2). In contrast, we found a 17% (HR 1.17; 95% CI 1.05–1.30) higher rate of all-cause mortality among men with a weight loss of 5 to 10 kg and a 55% (HR 1.55; 95% CI 1.31–1.84) higher rate of all-cause mortality among men with a weight loss > 10 kg. After excluding the first 2 years of follow-up the associations remained similar (Table S1). Results from models based on multiple imputation showed similar directions (Table S3). Finally, the estimated Sub-distribution Hazard Ratio (SHR) were lower, but confirmed the results obtained from the Cox model (Table S5 and S6).

### Weight loss and cause-specific mortality

We found a 19% (HR 1.19; 95% CI 1.04–1.37) higher rate of CVD mortality among those with a weight loss of 5 to 10 kg and a 27% (HR 1.27; 95% CI 1.01–1.59) higher rate following a weight loss of > 10 kg (Table 3). Further, in analyses stratified by sex, we found a 55% (HR 1.55; 95% CI 1.11- 2.15) higher rate of CVD mortality among men with a weight loss of > 10 kg but no association (HR 1.19; 95% CI 0.97–1.46) in men who lost 5 to 10 kg compared to those with no weight loss. In women, we found no association between weight loss of 5 to 10 kg or between weight loss of > 10 kg and CVD mortality (Table 3).

**Table 3:** Mortality rates and hazard ratios for the association between weight loss and cardiovascular-, and cancer mortality.

	Weight loss			*P* trend
	No weight loss	5–10 kg	> 10 kg	
Cardiovascular mortality				
Total				
Deaths (n)	957	490	149	
Person-years	331,808	212,947	78,722	
Crude mortality rate per 100,000 person-years	288.4	230.1	189.3	
HR (95% CI)^a^	1.00 (reference)	1.25 (1.12–1.39)	1.42 (1.19–1.69)	< 0.001
HR (95% CI)^b^	1.00 (reference)	1.19 (1.04–1.37)	1.27 (1.01–1.59)	0.005
Female				
Deaths (n)	419	278	85	
Person-years	199,172	153,054	61,547	
Crude mortality rate per 100,000 person-years	210.4	181.6	138.1	
HR (95% CI)^c^	1.00 (reference)	1.22 (1.05–1.43)	1.32 (1.04–1.67)	0.002
HR (95% CI)^d^	1.00 (reference)	1.18 (0.97–1.43)	1.11 (0.82–1.51)	0.20
Male				
Deaths (n)	538	212	64	
Person-years	132,637	59,893	17,175	
Crude mortality rate per 100,000 person-years	405.6	354.0	372.6	
HR (95% CI)^c^	1.00 (reference)	1.27 (1.08–1.49)	1.57 (1.21–2.04)	< 0.001
HR (95% CI)^d^	1.00 (reference)	1.19 (0.97–1.46)	1.55 (1.11–2.15)	0.005
Cancer mortality				
Total				
Deaths (n)	1029	560	194	
Person-years	331,808	212,947	78,722	
Crude mortality rate per 100,000 person-years	310.1	263.0	246.4	
HR (95% CI)^a^	1.00 (reference)	1.10 (0.99–1.22)	1.17 (1.00–1.36)	0.02
HR (95% CI)^b^	1.00 (reference)	1.05 (0.93–1.19)	1.04 (0.86–1.26)	0.50
Female				
Deaths (n)	462	369	133	
Person-years	199,172	153,054	61,547	
Crude mortality rate per 100,000 person-years	232.0	241.1	216.1	
HR (95% CI)^c^	1.00 (reference)	1.19 (1.04–1.36)	1.17 (0.97–1.42)	0.02
HR (95% CI)^d^	1.00 (reference)	1.04 (0.88–1.22)	0.96 (0.75–1.22)	0.93
Male				
Deaths (n)	567	191	61	
Person-years	132,637	59,893	17,175	
Crude mortality rate per 100,000 person-years	427.5	318.9	355.2	
HR (95% CI)^c^	1.00 (reference)	0.98 (0.83–1.15)	1.20 (0.92–1.57)	0.42
HR (95% CI)^d^	1.00 (reference)	1.06 (0.88–1.29)	1.23 (0.90–1.68)	0.20

*HR* hazard ratio, *CI* confidence interval.

^a^Adjusted for age and sex at enrollment.

^b^Adjusted for age, sex, body mass index, cigarette smoking, alcohol consumption, level of education, income, physical activity, sleep, and subjective health.

^c^Adjusted for age at enrollment.

^d^Adjusted for age, body mass index, cigarette smoking, alcohol consumption, level of education, income, physical activity, sleep, and subjective health.

We found no association in men or women between weight loss of 5 to 10 kg (HR 1.05; 95% CI 0.93–1.19), weight loss of > 10 kg (HR 1.04; 95% CI 0.86–1.26) and cancer mortality (Table 3). When we excluded the first two years of follow-up, the association remained nearly the same for men (Table S1). Results from models based on multiple imputation showed that an association with increased CVD mortality was present for men in both categories. In women, the association remained the same in models based on multiple imputation (Table S3).

### Number of weight losses and mortality

When we analyzed the number of times of weight loss of at least 5 kg (Table 4), we found an 11% (HR 1.11; 95% CI 1.02–1.20) higher rate of all-cause mortality among participants who reported weight loss once, a 16% (HR 1.16; 95% CI 1.03–1.30) higher rate of all-cause mortality for those who reported weight loss three times or more, compared to those who had never lost weight.

**Table 4:** Number of times of weight loss of ≥ 5 kg and hazard ratios of overall mortality, cardiovascular mortality, and cancer mortality.

Weight loss	Number of times	Number of subjects	Number of cases^a^	Crude HR^a^ 95% Cl	Number of cases^b^	Adjusted HR^b^ 95% Cl
All-cause mortality						
Number of times of weight loss ≥ 5 kg	0^c^	18,370	3333	1.00 (reference)	2379	1.00 (reference)
	1	8176	1193	1.13 (1.06–1.21)	856	1.11 (1.02–1.20)
	2	3805	556	1.15 (1.05–1.26)	408	1.10 (0.99–1.23)
	3+	3995	545	1.34 (1.22–1.47)	394	1.16 (1.03–1.30)
*P* trend				< 0.001		0.003
Cardiovascular mortality						
Number of times of weight loss ≥ 5 kg	0^c^	18,370	957	1.00 (reference)	634	1.00 (reference)
	1	8176	344	1.23 (1.09–1.39)	227	1.18 (1.01–1.38)
	2	3805	149	1.19 (1.00–1.42)	96	1.07 (0.85–1.33)
	3+	3995	146	1.58 (1.32–1.88)	114	1.49 (1.20–1.85)
*P* trend				< 0.001		0.001
Cancer mortality						
Number of times of weight loss ≥ 5 kg	0^c^	18,370	1,029	1.00 (reference)	779	1.00 (reference)
	1	8176	375	1.06 (0.94–1.20)	288	1.04 (0.91–1.19)
	2	3805	201	1.21 (1.04–1.41)	159	1.17 (0.98–1.39)
	3+	3995	178	1.13 (0.96–1.33)	126	0.93 (0.76–1.14)
*P* trend				0.02		0.78

*HR* hazard ratio, *CI* confidence interval.

^a^Adjusted for age and sex at enrollment.

^b^Adjusted for age, sex, body mass index, cigarette smoking, alcohol consumption, level of education, income, physical activity, sleep, and subjective health.

^c^Participants who answered “no” to the question “Have you ever lost 5 kg or more in less than a year?”.

For CVD mortality we found an 18% (HR 1.18; 95% CI 1.01–1.38) higher rate among those who had lost 5 kg or more at least once, a 49% (HR 1.49; 95% CI 1.20–1.85) higher rate among participants who had lost weight three times or more, compared to those who had never lost weight (Table 4). The association remained similar when we excluded the first two years of follow-up (Table S2) and used models based on multiple imputation (Table S4).

In contrast, we found no association between the number of times of weight loss of ≥ 5 kg and cancer mortality (Table 4). Results remained similar when we excluded the first two years of follow-up (Tables S1 and S2) and fitted models based on multiple imputation (Tables S3 and S4).

## Discussion

In this large prospective study, repeated weight loss of at least 5 kg three times or more was associated with increased all-cause and CVD mortality. A weight loss of more than 10 kg was associated with a 55% higher risk of both all-cause and CVD mortality in men with evidence of dose–response relationships both for amount and number of episodes of weight loss. In contrast, no statistically significant association was found in women. Further, we found no compelling association between weight loss and cancer mortality in men or women.

In our study, 48.6% reported that they had lost weight twice or more. Also in the National Health and Nutrition Examination Survey (2013–2016) in the United States, almost half of the respondents had tried to lose weight during the last year^16^. However, few people who intentionally lose weight are able to maintain the lost weight in the long term^17^. A meta-analysis showed indeed that 80% regain their lost weight within five years^18^.

Results from our study indicated that participants who had lost > 10 kg or at least 5 kg three times or more had the highest rate of all-cause and CVD mortality, which is in line with previous research showing associations between weight change and higher rates for all-cause^10–12^, as well as CVD mortality^10,12^. Similar to our study the Erfurt Male Cohort Study (ERFORT Study)^19^, comprising 1160 middle-aged men with a follow-up of 15 years, also showed that repeated weight changes were associated with increased mortality whilst the Nurses’ Health Study (NHS)^13^, a cohort comprising 44,876 women (middle-aged and older) also showed no association between weight cycling and all-cause mortality or CVD mortality. The European Prospective Investigation into Cancer in Norfolk (EPIC-Norfolk) cohort study^20^ with 15 years of follow-up reported that men who lost > 5 kg had higher all-cause and CVD mortality compared to those men who were weight stable. In contrast to our study, they found a higher rate of all-cause and CVD mortality, even among women who had lost > 5 kg compared to weight-stable women^20^.

The risk factors for CVD are similar for women and men. Still, men seem to develop cardiovascular diseases in earlier stages of life than women^21^. Differences in sex-specific hormones may impact cardiovascular health^22^. For instance, estrogens might protect women from CVD^22^. However, cardiovascular, and metabolic health depend on many interacting factors, where weight may be one.

In line with Zou et al.^10^ and Stevens et al.^14^, we found no association between repeated weight loss and cancer mortality. However, excess body weight itself has been associated with increased risk for several cancer sites^23^ and increased mortality in patients with a cancer diagnosis^24^. Therefore, weight loss might decrease cancer incidence and not contribute to increased cancer mortality. Furthermore, reduced cancer incidence has been reported among patients with obesity and diabetes following bariatric surgery during follow-up of more than 30 years^25^.

The strength of our prospective study is the large number of participants, and the long, virtually complete follow-up. Additionally, the linkage to the Swedish National Registers provided information with high validity about emigration, dates of death, and causes of death. However, despite the large number of participants in the cohort, the numbers of outcomes were small when stratifying into categories of number of times of weight loss and cause-specific mortality. Thus, we were not able to also conduct separate analyses for men and women. Another limitation in our study concerns the reference category “no weight loss”. We cannot exclude that this group might also include individuals who had gained, yet never lost, weight. Weight gain is a risk factor for many diseases^26^, and a high BMI is associated with increased mortality^27,28^. It is also possible that the significant association found in our study was due to weight fluctuation, not simply due to weight loss. During the long follow-up time, some participants may also have been exposed to weight changes after baseline. However, if present, this would most likely lead to underestimation of the excess risk.

A major limitation of our study was that it was unclear whether weight loss was voluntary or not. Although we excluded those with existing CVD and cancer diagnoses at the enrollment and performed sensitivity analyses to prevent reverse causality by excluding the first two years of follow-up results remained the same.

Another limitation was that we analyzed self-reported data collected at the study baseline. It can be challenging to remember the exact details concerning weight loss, such as the amount of weight loss and the number of weight losses. There is also a general concern that weight may be underreported due to social desirability^29^. It would have been preferable with objective anthropometric measurements, however, given the large study population, this was not feasible. However, self-reported weight and height data can be seen as valid measures in both men and women in epidemiologic studies^30,31^. We also acknowledge that residual confounding is a concern in any observational study. Although we extensively adjusted for potentially confounding factors, we may not have considered all possible confounders that were of importance, and we adjusted for factors at baseline, potentially years after the weight loss occurred. For instance, it would have been beneficial to adjust for pre-weight loss BMI and waist circumference, since weight loss may be beneficial in some cases while it may reflect an underlying disease in others. Further, smoking is a critical confounder strongly associated with mortality^32^. We adjusted for smoking, but since a large proportion of participants were former smokers, it could have been favorable to adjust for years since quitting. However, we lacked data on when participants quitted smoking. Further, the dose of smoking among current smokers may have an impact, but few participants in our cohort reported that they smoked more than 20 cigarettes per day. Additionally, the Swedish National March Cohort participants smoked less than the average Swede at that time (9.6% vs. 20.0%)^33,34^. They were instead more likely to have overweight and obesity compared to the Swedish population at the end of the 90’s (43.0% vs. 40.0%)^33,34^, while we know that those who smoke tend to have a lower body weight^35^. Another limitation of the generalizability is that barely 7% of the participants had obesity (BMI ≥ 30), making it difficult to conclusively examine whether the baseline BMI modifies the impact of weight loss.

This large epidemiological cohort gave us an opportunity to study the association between weight loss and mortality with more than 20 years of follow-up time and linkage to well-validated national registries. Our findings also articulate the still limited and inconsistent evidence that weight loss is causally associated with mortality. They also define several important issues that deserve further investigation: Is the apparent difference between women and men real or due to residual confounding; does baseline BMI mortality modify effects of weight loss; are effects of weight loss, at least in part, attributable to subsequent weight gain; and why is a sustained weight loss following bariatric surgery so convincingly associated with reduced mortality? Therefore, future analytical studies should focus on repeated objective weight measurements, more precise information on the time since weight loss and weight gain, as well as the identification of underlying causes of weight loss.

In conclusion, we found a significant association between previous weight loss, repeated weight loss and increased all-cause and CVD mortality in men, but not in women. We found no association with cancer mortality. Due to the high and ever-increasing prevalence of overweight and obesity, any causal association between weight loss and mortality would have substantial public health implications.

## Methods

### Study cohort

In September 1997, the Swedish Cancer Society organized a fund-raising event, the Swedish National March, in 3600 villages and cities around Sweden. Participants were invited to complete a detailed 36-page questionnaire about their lifestyle and medical history, including questions about height, weight, waist circumference, and if they had previously lost weight. The established cohort included 43,865 participants. The study design has been described in detail previously^33^.

**Figure 1:**
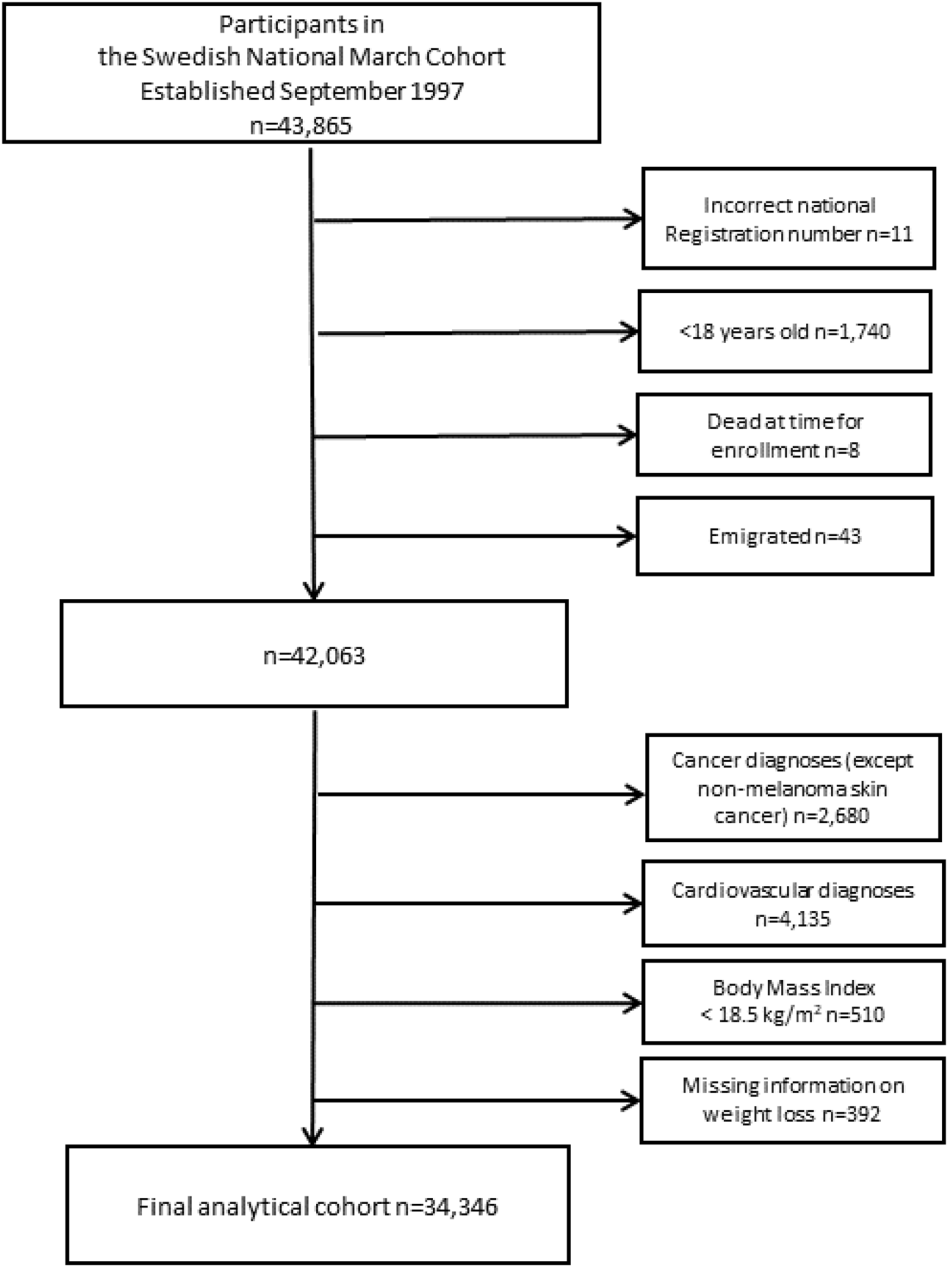
A flow-chart of study sample baseline exclusions and building the final analytical cohort.

Before the start of follow-up, we excluded individuals who had reported an incorrect national registration number (n = 11) or were < 18 years old (n = 1740) or had died (n = 8), or emigrated (n = 43), resulting in a cohort of 42,063 (Fig. 1). Further, according to the International Classifications (ICD) versions 7 to 10 through linkage with National inpatient and Outpatient Register (ICD-7: 330–334; 400–468; ICD-8: 390–459; ICD-9: 390–459; ICD-10: 100–199) we excluded participants with any history of CVD (n = 4135) and who had ever been diagnosed with cancer (n = 2680), except non-melanoma skin cancer through linkage with the National Cancer Register (ICD-coding for non-melanoma skin cancer: ICD-7:191). In addition, we excluded participants with underweight (BMI < 18.5 kg/m^2^) (n = 510) and with missing information on weight loss (n = 392). The final analytical cohort consisted of 34,346 participants.

### Exposure assessment

Weight loss was assessed from the questions “Have you ever lost 5 kg or more in less than a year?” with the answer options No/Yes. Respondents who replied “Yes” were further asked to report if the weight loss was ”between 5 and 10 kg” or “ > 10 kg”. Respondents were also asked to report how many times they had lost these amounts of weight, with response alternatives of 1, 2, 3, 4, or 5 times or more. Women were asked to disregard pregnancy-related weight changes.

Participants were divided into three weight loss categories: no weight loss, 5 to 10 kg, and > 10 kg. The category no weight loss included those who answered “No” to the question if they ever had lost 5 kg or more. The category 5 to 10 kg included those who lost, at least once, between 5 and 10 kg, but never > 10 kg. The category > 10 kg included those who lost more than 10 kg at least once. Participants were divided into four categories according to the number of times of weight loss: 0, 1, 2, and ≥ 3.

### Follow-up and outcomes

Participants were followed from October 1st, 1997, until the date of death, emigration, or end of follow-up in April 2018, whichever occurred first. Information on causes of death was available until December 2016, which thus became the end of follow-up in the cause-specific mortality analysis. At baseline, participants provided their individually unique national registration number, which allowed linkage to well-validated national registers. We used the Total Population Register, and the Migration Register to obtain dates of death, relocation, and emigration, the National Cause of Death Register to obtain causes of death, the National Patient Register and the National Cancer Register to obtain information on prevalent cancer and CVD cases, and the Longitudinal Integrated Database for Health Insurance and Labour Market Studies Database (LISA) to obtain data on education and income.

### Statistical analyses

Baseline characteristics of the study participants are reported over categories of weight loss. Categorical variables are summarized using absolute numbers and percentages, whereas continuous variables using mean and standard deviation (SD).

We fitted multivariable-adjusted Cox Proportional Hazards models with attained age as the underlying timescale to estimate Hazard Ratios (HRs) and 95% Confidence Intervals (CI) of the association between weight loss (both in terms of the amount of weight loss and frequency of weight loss) and all-cause, CVD, and cancer mortality. Cause-specific mortality was computed using cause-specific hazards for its straightforward interpretation. Due to the presence of competing risks, however, the cause-specific HRs do not carry a one-to-one correspondence of covariate effects on the cumulative incidence functions that characterize cause-specific mortality; therefore we estimated the Subdistribution Hazard Ratios (SHRs)^36^. As a reference group, we selected those who had never lost 5 kg or more in one year. These participants will be referred to as those with no weight loss.

Based on a priori assumptions, we included the following variables as potential confounders: sex, BMI, cigarette smoking, alcohol consumption, level of education, income, physical activity, sleep, and subjective health. BMI was categorized into ≤ 24.9, 25–29.9, and ≥ 30 kg/m^2^. We could not include waist circumference as a confounder due to a large number of missing values (25.1%). Smoking status was categorized as never, former, or current. Alcohol consumption was divided into none: reported never drinking alcohol, low: reported drinking alcohol less than four times/month, medium: reported drinking alcohol less than seven times/week, and high: reported drinking alcohol at least once a day. Due to low numbers, we combined medium and high alcohol consumption in one category. Education levels were categorized into compulsory school, high school, and university/Ph.D., and income were categorized in tertiles according to information obtained from the LISA database. Physical activity was categorized in sex-specific tertiles of total physical activity Metabolic Energy Turnover, MET-hours/day. Sleep duration was divided into ≤ 6, 7, and ≥ 8 h. Subjective health status was categorized into good/very good, neither good nor bad, and bad/very bad. We fitted models adjusted for age and sex and models additionally adjusted for all confounders listed above. Finally, we performed subgroup analyses (together with a formal test for multiplicative interaction) to assess the role of weight loss among women and men separately.

The Cox Proportional Hazards assumption was evaluated by testing the dependence of Schoenfeld’s residuals on time. No departure from proportionality was detected in all-cause mortality analyses. However, in cause-specific analyses, the variable sex did not fulfill the assumption, and therefore stratified Cox models on sex were implemented.

Participants who died in the first 2 years of follow-up might have had undiagnosed diseases when start of follow-up began, leading to unintentional weight loss. Therefore, we performed sensitivity analyses to prevent reverse causality, excluding the first 2 years of follow-up. Additionally, due to the presence of missing values, ranging from 0.1% (income) to 7.7% (smoking) on the covariates included in the models, we performed multiple imputation using the Multiple Imputation by Chained Equations algorithm, assuming a missing at random mechanism. We analyzed each imputed data set individually. Then we pooled the estimates to get the mean and the variance using Rubin’s method^37^. Twenty imputed datasets were thus created and analyzed. Analyses were performed using Stata, version 17 (StataCorp LLC, College Station, Texas). All statistical tests were two-sided, and p-values less than 0.05 were considered statistically significant.

This study was performed in the line with the principles of the Helsinki Declaration. Approval was granted by the Regional Ethical Review Committee in Stockholm (Dnr: 97-205 and 2017/796-31).

### Consent to participate

Informed consent was obtained from all individual participants included in the study.

## Supplementary Information

Supplementary Tables.

## Data Availability

The datasets analyzed during the current study are not publicly available due to ethical restrictions but are available from RB on reasonable request.
