# Supplementary material for: Prospective study of weight loss and all-cause-, cardiovascular-, and cancer mortality
Source: Sci Rep. 2023 Apr 6;13:5669. doi: 10.1038/s41598-023-32977-8 (PMC10079666; doi:10.1038/s41598-023-32977-8)
Supplement: Supplementary file 1 — Supplementary Tables. [file 41598_2023_32977_MOESM1_ESM.docx]

# **Supplementary information**

# Prospective study of weight loss and all-cause -, cardiovascular-, and cancer mortality

Liisa Tolvanen^1,2^*, Francesca Ghilotti^1^, Hans-Olov Adami^3,4^, Weimin Ye^3^, Stephanie E. Bonn^1^, Rino Bellocco^3,5^ and Ylva Trolle Lagerros^1,2^

1* Division of Clinical Epidemiology, Department of Medicine Solna, Karolinska Institutet, Stockholm, Sweden

2 Center for Obesity, Academic Specialist Center, Stockholm Health Services, Stockholm, Sweden

3 Department of Medical Epidemiology and Biostatistics, Karolinska Institutet, Stockholm, Sweden

4 Clinical Effectiveness Group, Institute of Health and Society, University of Oslo, Norway

5 Department of Statistics and Quantitative Methods, University of Milano-Bicocca, Milan, Italy

*Corresponding author. E-mail: [liisa.tolvanen@ki.se](mailto:liisa.tolvanen@ki.se)

**Supplement Figure Legends**

**Table S1.** Mortality rates and Hazard Ratios for the association between weight loss and overall mortality, cardiovascular mortality, and cancer mortality, excluding the first 2 years of follow-up

**Table S2.** Hazard Ratios for the association between number of times of weight loss (> 5 kg) and overall mortality, cardiovascular mortality, and cancer mortality after excluding the first 2 years of follow-up

**Table S3.** Hazard Ratios for the association between weight loss and overall mortality, cardiovascular mortality, and cancer mortality after carrying out multiple imputation (n = 34,346, females = 22,475, males = 11,871)

**Table S4.** Hazard Ratios for the association between number of times of weight loss (> 5 kg) and overall mortality, cardiovascular mortality, and cancer mortality after carrying out multiple imputation

**Table S5.** Mortality rates and Subdistribution Hazard Ratios for the association between weight loss and cardiovascular mortality and cancer mortality

**Table S6.** Number of times of weight loss of > 5 kg and Subdistribution Hazard Ratios of cardiovascular mortality, and cancer mortality

| **Table S1.** Mortality rates and Hazard Ratios for the association between weight loss and overall mortality, cardiovascular mortality, and cancer mortality, excluding the first 2 years of follow-up | | | | |
| --- | --- | --- | --- | --- |
|  | **Weight Loss** | | | ***P* trend** |
|  | **No weight loss** | **5-10 kg** | **>10 kg** |  |
| **All-cause mortality** |  |  |  |  |
| *Total* |  |  |  |  |
| Deaths (n) | 3,272 | 1,659 | 577 |  |
| Person-years | 314,917 | 202,732 | 75,017 |  |
| Crude mortality rate per 100,000 person-years | 1039.0 | 818.3 | 769.2 |  |
| HR (95% CI)^a^ | 1.00 (reference) | 1.12 (1.06-1.19) | 1.32 (1.20-1.44) | <0.001 |
| HR (95% CI)^b^ | 1.00 (reference) | 1.09 (1.01-1.17) | 1.20 (1.07-1.34) | 0.001 |
| *Female* |  |  |  |  |
| Deaths (n) | 1,519 | 1,004 | 364 |  |
| Person-years | 189,742 | 146,056 | 58,796 |  |
| Crude mortality rate per 100,000 person-years | 800.6 | 687.4 | 619.1 |  |
| HR (95% CI)^c^ | 1.00 (reference) | 1.10 (1.02-1.20) | 1.23 (1.10-1.38) | <0.001 |
| HR (95% CI)^d^ | 1.00 (reference) | 1.02 (0.93-1.13) | 1.03 (0.88-1.19) | 0.66 |
| *Male* |  |  |  |  |
| Deaths (n) | 1,753 | 655 | 213 |  |
| Person-years | 125,174 | 56,676 | 16,220 |  |
| Crude mortality rate per 100,000 person-years | 1400.4 | 1155.7 | 1313.2 |  |
| HR (95% CI)^c^ | 1.00 (reference) | 1.14 (1.04-1.25) | 1.48 (1.29-1.71) | <0.001 |
| HR (95% CI)^d^ | 1.00 (reference) | 1.16 (1.04-1.29) | 1.53 (1.28-1.82) | <0.001 |
| **Cardiovascular mortality** |  |  |  |  |
| *Total* |  |  |  |  |
| Deaths (n) | 938 | 475 | 146 |  |
| Person-years | 295,169 | 189,642 | 70,161 |  |
| Crude mortality rate per 100,000 person-years | 317.8 | 250.5 | 208.1 |  |
| HR (95% CI)^a^ | 1.00 (reference) | 1.23 (1.10-1.38) | 1.42 (1.19-1.69) | <0.001 |
| HR (95% CI)^b^ | 1.00 (reference) | 1.19 (1.04-1.37) | 1.25 (1.00-1.57) | 0.007 |
| *Female* |  |  |  |  |
| Deaths (n) | 414 | 274 | 83 |  |
| Person-years | 177,513 | 136,483 | 54,924 |  |
| Crude mortality rate per 100,000 person-years | 233.2 | 200.8 | 151.1 |  |
| HR (95% CI)^c^ | 1.00 (reference) | 1.22 (1.05-1.42) | 1.29 (1.02-1.64) | 0.004 |
| HR (95% CI)^d^ | 1.00 (reference) | 1.19 (0.98-1.44) | 1.08 (0.79-1.47) | 0.25 |
| *Male* |  |  |  |  |
| Deaths (n) | 524 | 201 | 63 |  |
| Person-years | 117,656 | 53,159 | 15,237 |  |
| Crude mortality rate per 100,000 person-years | 445.4 | 378.1 | 413,5 |  |
| HR (95% CI)^c^ | 1.00 (reference) | 1.24 (1.05-1.46) | 1.60 (1.23-2.08) | <0.001 |
| HR (95% CI)^d^ | 1.00 (reference) | 1.18 (0.96-1.45) | 1.58 (1.13-2.21) | 0.005 |
| **Cancer mortality** |  |  |  |  |
| *Total* |  |  |  |  |
| Deaths (n) | 1,000 | 545 | 185 |  |
| Person-years | 295,169 | 189,642 | 70,161 |  |
| Crude mortality rate per 100,000 person-years | 338.8 | 287.4 | 263.7 |  |
| HR (95% CI)^a^ | 1.00 (reference) | 1.09 (0.98-1.21) | 1.13 (0.96-1.32) | 0.06 |
| HR (95% CI)^b^ | 1.00 (reference) | 1.05 (0.93-1.19) | 1.01 (0.84-1.23) | 0.65 |
| *Female* |  |  |  |  |
| Deaths (n) | 448 | 359 | 127 |  |
| Person-years | 177,513 | 136,483 | 54,924 |  |
| Crude mortality rate per 100,000 person-years | 252.4 | 263.0 | 231.2 |  |
| HR (95% CI)^c^ | 1.00 (reference) | 1.18 (1.03-1.36) | 1.14 (0.93-1.39) | 0.046 |
| HR (95% CI)^d^ | 1.00 (reference) | 1.03 (0.87-1.21) | 0.93 (0.73-1.19) | 0.76 |
| *Male* |  |  |  |  |
| Deaths (n) | 552 | 186 | 58 |  |
| Person-years | 117,656 | 53,159 | 15,237 |  |
| Crude mortality rate per 100,000 person-years | 469.2 | 349.9 | 380.6 |  |
| HR (95% CI)^c^ | 1.00 (reference) | 0.97 (0.82-1.15) | 1.16 (0.88-1.52) | 0.59 |
| HR (95% CI)^d^ | 1.00 (reference) | 1.07 (0.88-1.29) | 1.20 (0.87-1.65) | 0.50 |
| *HR* Hazard Ratio, *CI* Confidence Interval  ^a^ Adjusted for age and sex at enrollment  ^b^ Adjusted for age, sex, body mass index, cigarette smoking, alcohol consumption, level of education, income, physical activity, sleep, and subjective health  ^c^ Adjusted for age at enrollment  ^d^ Adjusted for age, body mass index, cigarette smoking, alcohol consumption, level of education, income, physical activity, sleep, and subjective health | | | | |

|  |
| --- |

| **Table S2.** Hazard Ratios for the association between number of times of weight loss (>5 kg) and overall mortality, cardiovascular mortality, and cancer mortality after excluding the first 2 years of follow-up | | | | | | |
| --- | --- | --- | --- | --- | --- | --- |
| **Weight loss** | Number of  times | Number of subjects | Number of  cases^a^ | Crude HR^a^  95% Cl | Number of cases^b^ | Adjusted HR ^b^ 95% Cl |
| **All-cause mortality** |  |  |  |  |  |  |
| Number of times | 0^c^ | 18,250 | 3,272 | 1.00 (reference) | 2,335 | 1.00 (reference) |
| of weight loss >5 kg | 1 | 8,120 | 1,161 | 1.12 (1.05-1.20) | 835 | 1.10 (1.01-1.19) |
|  | 2 | 3,786 | 548 | 1.14 (1.05-1.25) | 405 | 1.11 (1.00-1.24) |
|  | 3+ | 3,972 | 527 | 1.31 (1.19-1.44) | 379 | 1.12 (1.00-1.27) |
| ***P* trend** |  |  |  | <0.001 |  | 0.009 |
| **Cardiovascular mortality** |  |  |  |  |  |  |
| Number of times | 0^c^ | 18,250 | 938 | 1.00 (reference) | 618 | 1.00 (reference) |
| of weight loss >5 kg | 1 | 8,120 | 333 | 1.22 (1.07-1.38) | 223 | 1.19 (1.01-1.39) |
|  | 2 | 3,786 | 148 | 1.20 (1.01-1.43) | 95 | 1.07 (0.86-1.34) |
|  | 3+ | 3,972 | 140 | 1.54 (1.28-1.84) | 109 | 1.45 (1.16-1.81) |
| ***P* trend** |  |  |  | <0.001 |  | 0.002 |
| **Cancer mortality** |  |  |  |  |  |  |
| Number of times | 0^c^ | 18,250 | 1,000 | 1.00 (reference) | 759 | 1.00 (reference) |
| of weight loss >5 kg | 1 | 8,120 | 364 | 1.05 (0.93-1.19) | 280 | 1.03 (0.90-1.19) |
|  | 2 | 3,786 | 197 | 1.21 (1.04-1.42) | 158 | 1.18 (0.99-1.42) |
|  | 3+ | 3,972 | 169 | 1.09 (0.92-1.28) | 119 | 0.89 (0.73-1.10) |
| ***P* trend** |  |  |  | 0.05 |  | 0.96 |
| *HR* Hazard Ratio, *CI* Confidence Interval  ^a^ Adjusted for age and sex at enrollment  ^b^ Adjusted for age, sex, body mass index, cigarette smoking, alcohol consumption, level of education, income, physical activity, sleep, and subjective health  ^c^ Participants who answered “no” to the question “Have you ever lost 5 kg or more in less than a year?” | | | | | | |

| **Table S3.** Hazard Ratios for the association between weight loss and overall mortality, cardiovascular mortality, and cancer mortality after carrying out multiple imputation (n = 34,346, females = 22,475, males = 11,871) | | | | |
| --- | --- | --- | --- | --- |
|  | **Weight Loss** | | | ***P* trend** |
|  | **No weight loss** | **5-10 kg** | **>10 kg** |  |
| **All-cause mortality** |  |  |  |  |
| *Total* |  |  |  |  |
| HR (95% CI)^a^ | 1.00 (reference) | 1.13 (1.07-1.20) | 1.34 (1.23-1.47) | <0.001 |
| HR (95% CI)^b^ | 1.00 (reference) | 1.06 (1.00-1.13) | 1.17 (1.07-1.29) | 0.001 |
| *Female* |  |  |  |  |
| HR (95% CI)^a^ | 1.00 (reference) | 1.11 (1.02-1.20) | 1.26 (1.12-1.41) | <0.001 |
| HR (95% CI)^b^ | 1.00 (reference) | 1.03 (0.95-1.12) | 1.09 (0.96-1.23) | 0.17 |
| *Male* |  |  |  |  |
| HR (95% CI)^a^ | 1.00 (reference) | 1.16 (1.06-1.26) | 1.51 (1.31-1.74) | <0.001 |
| HR (95% CI)^b^ | 1.00 (reference) | 1.10 (1.00-1.20) | 1.35 (1.17-1.57) | <0.001 |
| **Cardiovascular mortality** |  |  |  |  |
| *Total* |  |  |  |  |
| HR (95% CI)^a^ | 1.00 (reference) | 1.25 (1.12-1.39) | 1.42 (1.19-1.69) | <0.001 |
| HR (95% CI)^b^ | 1.00 (reference) | 1.15 (1.03-1.29) | 1.21 (1.01-1.45) | 0.006 |
| *Female* |  |  |  |  |
| HR (95% CI)^c^ | 1.00 (reference) | 1.22 (1.05-1.43) | 1.32 (1.04-1.67) | 0.002 |
| HR (95% CI)^d^ | 1.00 (reference) | 1.12 (0.96-1.31) | 1.12 (0.87-1.43) | 0.18 |
| *Male* |  |  |  |  |
| HR (95% CI)^c^ | 1.00 (reference) | 1.27 (1.08-1.49) | 1.57 (1.21-2.04) | <0.001 |
| HR (95% CI)^d^ | 1.00 (reference) | 1.19 (1.01-1.40) | 1.36 (1.04-1.78) | 0.006 |
| **Cancer mortality** |  |  |  |  |
| *Total* |  |  |  |  |
| HR (95% CI)^a^ | 1.00 (reference) | 1.10 (0.99-1.22) | 1.17 (1.00-1.36) | 0.02 |
| HR (95% CI)^b^ | 1.00 (reference) | 1.04 (0.93-1.16) | 1.05 (0.89-1.24) | 0.47 |
| *Female* |  |  |  |  |
| HR (95% CI)^c^ | 1.00 (reference) | 1.19 (1.04-1.36) | 1.17 (0.97-1.42) | 0.02 |
| HR (95% CI)^d^ | 1.00 (reference) | 1.12 (0.97-1.29) | 1.04 (0.84-1.28) | 0.37 |
| *Male* |  |  |  |  |
| HR (95% CI)^c^ | 1.00 (reference) | 0.98 (0.83-1.15) | 1.20 (0.92-1.57) | 0.42 |
| HR (95% CI)^d^ | 1.00 (reference) | 0.93 (0.78-1.10) | 1.12 (0.85-1.48) | 0.95 |
| *HR* Hazard Ratio, *CI* Confidence Interval  ^a^ Adjusted for age and sex at enrollment  ^b^ Adjusted for age, sex, body mass index, cigarette smoking, alcohol consumption, level of education, income, physical activity, sleep, and subjective health  ^c^ Adjusted for age at enrollment  ^d^ Adjusted for age, body mass index, cigarette smoking, alcohol consumption, level of education, income, physical activity, sleep, and subjective health | | | |  |

| **Table S4.** Hazard Ratios for the association between number of times of weight loss (> 5 kg) and overall mortality, cardiovascular mortality, and cancer mortality after carrying out multiple imputation. | | | |
| --- | --- | --- | --- |
| **Weight loss** | Number of times | Crude HR^a^ 95% Cl | Adjusted HR^b^ 95% Cl |
| **All-cause mortality** |  |  |  |
| Number of times | 0^c^ | 1.00 (reference) | 1.00 (reference) |
| of weight loss >5 kg | 1 | 1.13 (1.06-1.21) | 1.07 (1.00-1.15) |
|  | 2 | 1.15 (1.05-1.26) | 1.05 (0.96-1.15) |
|  | 3+ | 1.34 (1.22-1.47) | 1.17 (1.06-1.29) |
| ***P* trend** |  | <0.001 | 0.002 |
| **Cardiovascular mortality** |  |  |  |
| Number of times | 0^c^ | 1.00 (reference) | 1.00 (reference) |
| of weight loss >5 kg | 1 | 1.23 (1.09-1.39) | 1.16 (1.02-1.31) |
|  | 2 | 1.19 (1.00-1.42) | 1.07 (0.89-1.27) |
|  | 3+ | 1.58 (1.32-1.88) | 1.34 (1.11-1.61) |
| ***P* trend** |  | <0.001 | 0.003 |
| **Cancer mortality** |  |  |  |
| Number of times | 0^c^ | 1.00 (reference) | 1.00 (reference) |
| of weight loss >5 kg | 1 | 1.06 (0.94-1.20) | 1.01 (0.90-1.14) |
|  | 2 | 1.21 (1.04-1.41) | 1.13 (0.97-1.32) |
|  | 3+ | 1.13 (0.96-1.33) | 1.01 (0.85-1.20) |
| ***P* trend** |  | 0.02 | 0.45 |
| *HR* Hazard Ratio, *CI* Confidence Interval  ^a^ Adjusted for age and sex at enrollment  ^b^ Adjusted for age, sex, body mass index, cigarette smoking, alcohol consumption, level of education, income, physical activity, sleep, and subjective health  ^c^ Participants who answered “no” to the question “Have you ever lost 5 kg or more in less than a year?” | | | |

| **Table S5.** Mortality rates and Subdistribution Hazard Ratios for the association between weight loss and cardiovascular mortality and cancer mortality | | | | | |  | |
| --- | --- | --- | --- | --- | --- | --- | --- |
|  | **Weight Loss**  ***P* trend** | | | | | |  |
|  | **No weight loss** | **5-10 kg** | | **>10 kg** |  | |  |
| **Cardiovascular mortality** |  | |  |  |  | |  |
| *Total* |  | |  |  |  | |  |
| Deaths (n) | 957 | | 490 | 149 |  | |  |
| Person-years | 331,808 | | 212,947 | 78,722 |  | |  |
| Crude mortality rate per 100,000 person-years | 288.4 | | 230.1 | 189.3 |  | |  |
| SHR (95% CI)^a^ | 1.00 (reference) | | 1.20 (1.08-1.34) | 1.30 (1.09-1.54) | <0.001 | |  |
| SHR (95% CI)^b^ | 1.00 (reference) | | 1.18 (1.03-1.35) | 1.17 (0.93-1.47) | 0.03 | |  |
| *Female* |  | |  |  |  | |  |
| Deaths (n) | 419 | | 278 | 85 |  | |  |
| Person-years | 199,172 | | 153,054 | 61,547 |  | |  |
| Crude mortality rate per 100,000 person-years | 210.4 | | 181.6 | 138.1 |  | |  |
| SHR (95% CI)^c^ | 1.00 (reference) | | 1.16 (1.00-1.35) | 1.19 (0.95-1.50) | 0.03 | |  |
| SHR (95% CI)^d^ | 1.00 (reference) | | 1.17 (0.96-1.41) | 1.05 (0.77-1.43) | 0.34 | |  |
| *Male* |  | |  |  |  | |  |
| Deaths (n) | 538 | | 212 | 64 |  | |  |
| Person-years | 132,637 | | 59,893 | 17,175 |  | |  |
| Crude mortality rate per 100,000 person-years | 405.6 | | 354.0 | 372.6 |  | |  |
| SHR (95% CI)^c^ | 1.00 (reference) | | 1.23 (1.05-1.44) | 1.49 (1.15-1.94) | <0.001 | |  |
| SHR (95% CI)^d^ | 1.00 (reference) | | 1.17 (0.96-1.42) | 1.38 (0.99-1.93) | 0.03 | |  |
| **Cancer mortality** |  | |  |  |  | |  |
| *Total* |  | |  |  |  | |  |
| Deaths (n) | 1,029 | | 560 | 194 |  | |  |
| Person-years | 331,808 | | 212,947 | 78,722 |  | |  |
| Crude mortality rate per 100,000 person-years | 310.1 | | 263.0 | 246.4 |  | |  |
| SHR (95% CI)^a^ | 1.00 (reference) | | 1.07 (0.97-1.19) | 1.13 (0.97-1.32) | 0.07 | |  |
| SHR (95% CI)^b^ | 1.00 (reference) | | 1.03 (0.91-1.17) | 1.03 (0.85-1.24) | 0.67 | |  |
| *Female* |  | |  |  |  | |  |
| Deaths (n) | 462 | | 369 | 133 |  | |  |
| Person-years | 199,172 | | 153,054 | 61,547 |  | |  |
| Crude mortality rate per 100,000 person-years | 232.0 | | 241.1 | 216.1 |  | |  |
| SHR (95% CI)^c^ | 1.00 (reference) | | 1.18 (1.03-1.35) | 1.15 (0.95-1.40) | 0.03 | |  |
| SHR (95% CI)^d^ | 1.00 (reference) | | 1.02 (0.87-1.20) | 0.95 (0.74-1.21) | 0.83 | |  |
| *Male* |  | |  |  |  | |  |
| Deaths (n) | 567 | | 191 | 61 |  | |  |
| Person-years | 132,637 | | 59,893 | 17,175 |  | |  |
| Crude mortality rate per 100,000 person-years | 427.5 | | 318.9 | 355.2 |  | |  |
| SHR (95% CI)^c^ | 1.00 (reference) | | 0.94 (0.80-1.11) | 1.12 (0.86-1.46) | 0.85 | |  |
| SHR (95% CI)^d^ | 1.00 (reference) | | 1.04 (0.86-1.26) | 1.17 (0.85-1.60) | 0.36 | |  |
| *SHR* Subdistribution Hazard Ratio, *CI* Confidence Interval  ^a^ Adjusted for age and sex at enrollment  ^b^ Adjusted for age, sex, body mass index, cigarette smoking, alcohol consumption, level of education, income, physical activity, sleep, and subjective health  ^c^ Adjusted for age at enrollment  ^d^ Adjusted for age, body mass index, cigarette smoking, alcohol consumption, level of education, income, physical activity, sleep, and subjective health | | | | | |  | |

| **Table S6.** Number of times of weight loss of > 5 kg and Subdistribution Hazard Ratios of cardiovascular mortality, and cancer mortality | | | | | | | |
| --- | --- | --- | --- | --- | --- | --- | --- |
| **Weight loss** | Number of times | Number of subjects | Number of  cases^a^ | Crude SHR^a^ 95% Cl | Number of cases^b^ | Adjusted SHR ^b^ 95% Cl | |
| **Cardiovascular**  **mortality** |  |  |  |  |  |  | |
| Number of times | 0^c^ | 18,370 | 957 | 1.00 (reference) | 634 | 1.00 (reference) | |
| of weight loss >5 kg | 1 | 8,176 | 344 | 1.20 (1.06-1.36) | 227 | 1.16 (1.00-1.36) | |
|  | 2 | 3,805 | 149 | 1.14 (0.96-1.35) | 96 | 1.03 (0.82-1.29) | |
|  | 3+ | 3,995 | 146 | 1.40 (1.18-1.67) | 114 | 1.43 (1.15-1.78) | |
| ***P* trend** |  |  |  | <0.001 |  | 0.005 | |
| **Cancer mortality** |  |  |  |  |  |  | |
| Number of times | 0^c^ | 18,370 | 1,029 | 1.00 (reference) | 779 | 1.00 (reference) | |
| of weight loss >5 kg | 1 | 8,176 | 375 | 1.00 (0.89-1.12) | 288 | 1.02 (0.89-1.18) | |
|  | 2 | 3,805 | 201 | 1.12 (0.96-1.30) | 159 | 1.16 (0.97-1.38) | |
|  | 3+ | 3,995 | 178 | 1.01 (0.86-1.19) | 126 | 0.91 (0.74-1.11) | |
| ***P* trend** |  |  |  | 0.44 |  | 0.99 | |
| *SHR* Subdistribution Hazard Ratio, *CI* Confidence Interval  ^a^ Adjusted for age and sex at enrollment  ^b^ Adjusted for age, sex, body mass index, cigarette smoking, alcohol consumption, level of education, income, physical activity, sleep, and subjective health  ^c^ Participants who answered “no” to the question “Have you ever lost 5 kg or more in less than a year?” | | | | | | |  |
